# Protective Effect of Protocatechuic Acid on TNBS-Induced Colitis in Mice Is Associated with Modulation of the SphK/S1P Signaling Pathway

**DOI:** 10.3390/nu9030288

**Published:** 2017-03-16

**Authors:** Irene Crespo, Beatriz San-Miguel, José Luis Mauriz, Juan José Ortiz de Urbina, Mar Almar, María Jesús Tuñón, Javier González-Gallego

**Affiliations:** 1 Institute of Biomedicine (IBIOMED), University of León, 24071 León, Spain; icreg@unileon.es (I.C.); bsanv@unileon.es (B.S.-M.); jl.mauriz@unileon.es (J.L.M.); malmg@unileon.es (M.A.); mjtung@unileon.es (M.J.T.); 2 Centro de Investigación Biomédica en Red de Enfermedades Hepáticas y Digestivas (CIBERehd), 24071, Spain; 3 Pharmacy Service, Complejo Asistencial Universitario de León, 24071 León, Spain; jortiz@saludcastillayleon.es

**Keywords:** protocatechuic acid, colitis, inflammation, sphingolipids, SphK/S1P signaling

## Abstract

(1) Background: The present study aimed to investigate whether beneficial effects of protocatechuic acid (PCA) are associated with inhibition of the SphK/S1P axis and related signaling pathways in a 2,4,6-trinitrobenzenesulfonic acid (TNBS) model of inflammatory bowel disease; (2) Methods: Colitis was induced in male Balb/c mice by intracolonic administration of 2 mg of TNBS. PCA (30 or 60 mg/kg body wt) was given intraperitoneally daily for five days; (3) Results: Administration of PCA prevented the macroscopic and microscopic damage to the colonic mucosa, the decrease in body weight gain and the increase in myeloperoxidase activity induced by TNBS. PCA-treated mice exhibited a lower oxidized/reduced glutathione ratio, increased expression of antioxidant enzymes and Nrf2 and reduced expression of proinflammatory cytokines. Following TNBS treatment mRNA levels, protein concentration and immunohistochemical labelling for SphK1 increased significantly. S1P production and expression of S1P receptor 1 and S1P phosphatase 2 were significantly elevated. However, there was a decreased expression of S1P lyase. Furthermore, TNBS-treated mice exhibited increased phosphorylation of AKT and ERK, and a higher expression of pSTAT3 and the NF-κB p65 subunit. PCA administration significantly prevented those changes; (4) Conclusions: Data obtained suggest a contribution of the SphK/S1P system and related signaling pathways to the anti-inflammatory effect of PCA.

## 1. Introduction

Inflammatory bowel disease (IBD) is a chronic intestinal inflammatory disorder encompassing ulcerative colitis and Crohn’s disease. Though the etiology of IBD remains unknown, etiological factors possibly include genetic predisposition, immunological abnormalities and environmental factors [1]. Early in the disease, immunologic reactions promote release of different pro-inflammatory cytokines, such as interleukin (IL)-6 and tumor necrosis factor (TNF)-α. These mediators initiate pathways that activate key inflammatory transcription factors, including nuclear factor kappa B (NF-κB) and signal transducer and activator of transcription (STAT)3, resulting in the chronic tissue inflammation that characterizes human IBD [2]. Sphingosine-1-phosphate (S1P) is a pleiotropic bioactive lipid mediator formed by the phosphorylation of sphingosine by sphingosine kinases (SphK) which, after binding to S1P receptors (S1PR), activates different downstream signaling pathways and regulates an array of biological activities in various cell types [3,4]. S1P serves as a major activator of the STAT3 and NF-κB pathways implicated in the pathophysiology of IBD [2]. Different studies have shown that both SphK and S1P are upregulated in colonic tissues and S1P modulation shows effectiveness for alleviating multiple aspects of chronic intestinal inflammation [5,6]. Thus, targeting S1P signaling may represent a novel strategy in treating IBD.

Natural phenolic compounds found in edible fruits and vegetables have shown efficacy both in clinical trials and in experimental models of rodent colitis through different mechanisms including protection against oxidative stress and immunomodulatory properties [7]. Although very little is known regarding the extent to which oxidative stress impacts S1P metabolism, it has been reported that beneficial effects of the flavonoid resveratrol in experimental ulcerative colitis may be linked to its inhibitory effect on SphK1 [8]. Moreover, it has been hypothesized that oxidative stress could enhance inflammation in IBD and colitis-associated colon cancer through impaired S1P degradation [2]. Protocatechuic acid (PCA, 3-4-dihydroxybenzoic acid) is a simple phenolic acid found in many edible vegetables, fruits and nuts [9], which has been identified as a major metabolite of complex polyphenols and considered the best biomarker of flavonoid consumption [10,11]. A number of cellular and animal studies have shown that PCA has multifaceted biological effects, including a potent antioxidant and anti-inflammatory activity, in different tissues [9]. Recently, it has been reported that oral administration of PCA ameliorates dextran sulphate sodium (DSS)-induced IBD in rats, with inhibition of oxidative stress, increased activity of antioxidant enzymes and lowered expression of pro-inflammatory mediators [12]. The present study was undertaken to analyze whether beneficial effects of PCA are associated with inhibition of the SphK/S1P axis and related signaling pathways in a colitis mice model induced by 2,4,6-trinitrobenzenesulfonic acid (TNBS). Results obtained support the protective role of PCA on TNBS-induced colonic damage and reinforce the interest of the SphK/S1P system as an attractive target for therapeutic interventions in IBD.

## 2. Materials and Methods 

### 2.1. Animal Experiments and Drug Treatment

Male Balb/c mice (Harlan Laboratories; BCN, Spain) weighing 20–25 g were used in this study. The animals were acclimated to the temperature (22 ± 2 °C) and humidity (55% ± 5%) of controlled rooms with a 12- to 12-h light–dark cycle for at least a week prior to experiments. They were allowed access to mice chow and water ad libitum. Experimental colitis was induced by TNBS. Briefly, mice fasted for 24 h were lightly anesthetized with isoflurane, and a polyethylene catheter (2 mm in outer diameter) was inserted rectally until the splenic flexure (3.5–4 cm from the anus). Then, 2 mg of TNBS (Sigma Chemical Co., St. Louis, MO, USA) dissolved in a volume of 0.1 mL of ethanol 50% (*v*/*v*) were administered through the catheter. To distribute the agents within the entire colon, mice were held in a vertical position for 30 s after the injection. PCA (Sigma Chemical Co.) was dissolved into saline at two concentrations (30 mg/kg and 60 mg/kg), and administered by intraperitoneal route starting from the day of TNBS administration and continued daily up to the end of the study at day 5. The mice were randomly divided into 5 groups: a control group that received only vehicle (C), a group that received PCA 60 mg/kg (C + PCA), a colitic group that received TNBS (TNBS), and two additional colitic groups which received 30 or 60 mg of PCA (TNBS + PCA30 and TNBS + PCA60, respectively). Each group consisted of six mice. The study protocol was carried out in strict accordance with the recommendations in the Guide for the Care and Use of Laboratory Animals of the National Institutes of Health and was specifically approved by the Ethics Committee of the University of León (No. LE-2-00022-c02-02). At the end of the experiments, mice were anesthetized with ketamine/xylazine (Sigma Chemical Co.) cocktail and sacrificed. The colon was sectioned for 3 uses: (1) fixed with 10% buffered formalin for histologic examinations; (2) preserved at −80 °C for Western blot; and (3) homogenized in Trizol for RNA isolation.

### 2.2. Macroscopic and Microscopic Analysis

Macroscopic and microscopic damage of the colonic mucosa was assessed by two observers blinded to the treatment given, according to previously established scores [13]. The scale for macroscopic damage ranged from 0 to 4 as follows: 0 = normal appearance; 1 = mucosal erythema only; 2 = mild edema, slight bleeding or small erosions; 3 = moderate edema, bleeding, ulcers or erosions; 4 = severe ulcerations, erosions, edema, and tissue necrosis. Samples were rinsed with saline, fixed in 10% buffered formalin and embedded in paraffin blocks. Slices with 5 μm sections were stained with hematoxylin and eosin. Ten non-overlapping randomly chosen fields of each section were scored according to the following scale: 0 = infiltrated normocellular or normal hypercellular lamina, polymorphonuclear neutrophils (PMNs) absent; 1 = diffuse PMNs in lamina propria, occasional cryptitis but few cryptic abscesses, minimal glandular destruction or ulceration; 2 = moderate number of PMNs in lamina propria, cryptitis and prominent cryptic abscesses, some glandular destruction; 3 = numerous PMNs with abundant cryptitis, cryptic abscesses, extensive cellular destruction, prominent ulceration. 

### 2.3. Tissue Myeloperoxidase Activity

Myeloperoxidase (MPO) activity, which is reflective of neutrophil influx into the colon and often used as a marker on inflammation, was assayed spectrophotometrically [14]. Briefly, a portion of colon tissue was homogenized with a Polytron homogenizer, using the homogenization buffer (50 mM potassium phosphate, pH 6.0) containing 0.5% hexadecyltrimethyl ammonium bromide (Sigma Chemical Co.). The homogenates were subjected to sonication in an ice bath for 10 s (Heat Systems Ultrasonics, Plainview, NY, USA), after which they were freeze-thawed three times. Sonication was repeated, and the samples were centrifuged at 40,000× *g* for 15 min. MPO in supernatants was assayed with *o*-dianisidine dihydrochloride (Sigma Chemical Co.) and 0.0005% hydrogen peroxide (Mallinckrodt Inc., Paris, France) as the substrate. Every assay was performed in triplicate. Activity was assayed over 5 min at wavelength 460 nm (BioTek Synergy HT, Winooski, VT, USA) and expressed as units per mg of total protein (U/mg protein).

### 2.4. GSH and GSSG Analysis

The intracellular reduced (GSH) and oxidized glutathione (GSSG) content were measured as previously reported [15]. Briefly, 25 mg of colon tissue was homogenized in 0.1 M sodium phosphate 5 mM EDTA buffer (pH 8.0) with 25% phosphoric acid to precipitate protein. The mixture was centrifuged at 100,000× *g* for 30 min at 4 °C, the supernatant was collected and 500 µL were diluted with 4.5 mL of buffer. For GSH measurement, to 100 μL supernatant, 1.8 mL phosphate-EDTA buffer and 100 μL *o*-phthalaldehyde (50 μg/mL, Sigma Chemical Co.) were added and mixing for 15 min, then the fluorescence was detected at 420 nm (excitation at 350 nm). For GSSG assay, 500 µL of the sample supernatant was incubated with 200 µL of 0.04 M N-ethylmaleimide (Sigma Chemical Co.) for 30 min; to this mixture 4.5 mL of 0.1 N NaOH was added. A 100 µL portion of this mixture was then processed using the procedure outlined above for GSH assay. Every assay was performed in triplicate.

### 2.5. Real-Time Reverse Transcription-Polymerase Chain Reaction (RT-PCR) 

Total RNA was obtained from frozen mouse colon using a Trizol reagent (Life Technologies, Madrid, Spain) and quantified using a Nano Drop 1000 spectrophotometer (Thermo Scientific, Wilmington, DE, USA). Residual genomic DNA was removed by incubating RNA with RQ1 RNase-free DNase (Promega, Madison, WI, USA). RNA integrity was confirmed by formaldehyde gel electrophoresis. Total RNA (1 μg) was reverse transcribed as described [16] and mRNA was determined by real-time PCR analysis using SYBER Green I Master (Applied Biosystems, Foster City, CA) and the appropriate primers for SphK1 (5′ACTGATACTCACCGAACGGAA3′ and 5′CCATCACCGGACATGACTGC3′), S1PR1 (5′ATGGTGTCCACTAGCATCCC3′ and 5′CGATGTTCAACTTGCCTGTGTAG3′), S1PL (5′ACCAGACCCTTTCCCACATTT3′ and 5′ACTGCCCACATGTGCAGGAT3′), SGPP2 (5′CTCTGAGCCCAAGACAGTTAGGA3′ and 5′TCGACAGCGTTCTCCATTTTG3′), and β-Actin (5′AATCGTGCGTGACATCAAAGAG3′ and 5′GCCATCTCCTGCTCGAAGTCT3′) or TaqMan Gene Expression Assay (Applied Biosystems) and primers and probes for IL-6 (GenBank accession No. NM_031168.1 and Mm00446190_m1), TNF-α (GenBank accession No. NM_013693.3 and Mm00443258_m1), IL-1β (GenBank accession No. NM_008361.3 and Mm01336189_m1), COX-2 (GenBank accession No. NM_011198.3 and Mm00478374_m1) and β-Actin (GenBank accession No. EF095208.1 and Mm02619580_g1) genes. Relative changes in gene expression levels were determined using the 2^−ΔΔCt^ method. The cycle number of β-Actin gene detection, referred to as ΔCt.

### 2.6. Western Blot Analysis

For Western blot analysis, colon tissue (25 mg) was homogeneized in 1 mL radioimmunoprecipitation assay buffer (RIPA) containing protease and phosphatase inhibitor cocktails (Roche Diagnostics GmbH, Mannheim, Germany), maintaining temperature at 4 °C throughout all procedures. Then the homogenate was incubated on ice for 30 min and finally the samples were centrifuged at 13,000× *g* for 30 min at 4 °C. The supernatant fraction was stored at −80 °C in aliquots until use. Protein concentration was measured by Bradford assay. Equal amounts of protein extracts (25 μg) were separated by 9%–12% sodium dodecyl sulfate (SDS)-polyacrylamide gel electrophoresis and transferred electrically to polyvinylidene difluoride membranes (Millipore, Bedford, MA, USA). The membranes were then blocked with 5% nonfat dry milk in Tris-buffered saline containing 0.05% Tween 20 for 30 min at 37 °C and probed overnight at 4 °C with polyclonal SOD-1, CAT, Nrf2, phospho-AKT, AKT, phospho-ERK1/2, ERK and NF-κB p65 (Santa Cruz Biotechnology, Inc., Santa Cruz, CA, USA), SphK1 and S1PR1 (Abcam, Cambridge, UK), and phospho-STAT-3 and STAT-3 (Cell Signaling Technology, Danvers, MA, USA) antibodies at 1:200–1:1000 dilution with PBST containing 2.5% nonfat dry milk. Equal loading of protein was demonstrated by probing the membranes with a rabbit anti-β-actin polyclonal antibody (1:20,000; Sigma). After washing with Tris-buffered saline containing 0.05% Tween 20, the membranes were incubated for 1 h at room temperature with secondary horseradish peroxidase-conjugated antibody (1:5000; Dako, Glostrup, Denmark) and visualized using enhanced chemiluminescence (ECL) detection kit (Amersham Pharmacia, Uppsala, Sweden) [17]. Band intensities were quantified using the software Image J (NIH, Bethesda, MD, USA). Each assay was performed in triplicate.

### 2.7. Immunohistochemical Analysis 

Immunohistochemistry was performed using polyclonal antibody for SphK1 and COX-2. Tissue samples were recovered, fixed in 10% buffered formalin, and embedded in paraffin. Section (4 µm) were dewaxed and hydrated through graded ethanol, cooked in 25 mM citrate buffer, pH 6.0, in a pressure cooker for 10 min, transferred into boiling deionized water, and let to cool for 20 min. Tissue sections were then treated with 3% hydrogen peroxide to inactivate endogenous peroxidase activity. The slides were incubated with anti-SphK1 (Abcam) and anti-COX-2 (Cell Signaling) antibodies at a working dilution of 1:200 overnight at 4 °C [18] and a secondary polymer-based detection system (EnVision+ System Labelled Polymer-horseradish peroxidase anti-rabbit; Dako). The chromogen was 3,3′diaminobenzidine (DAB; Vector Laboratories, Burlingame, CA, USA) and sections were counterstained with haematoxylin. The specificity of technique was evaluated by negative controls (omitting the incubation with the primary antibody and incubating it with nonimmune sera). Areas of staining were analyzed by WinRoof version 6.3 (Mitani, Tokio, Japan) software with ten non-overlapping randomly chosen histological fields. Results were expressed as the percentage of stained cells in each field.

### 2.8. Assay for S1P Levels

S1P was tested by ELISA using a commercial kit (Echelon Biosciences, Salt Lake City, UT, USA). Quantitative S1P assays were done in triplicate on all colon tissue homogenate samples according to manufacturer’s instructions. The levels were expressed as pmol/μg of protein.

### 2.9. Statistical Analysis

Results are expressed as mean values ± standard error of the mean (SEM). Data were compared by analysis of variance (ANOVA); when the analysis indicated the presence of significant difference, the means were compared with the Newman-Keul’s test. Significance was accepted when *p* was < 0.05. Values were analyzed using the statistical package SPSS 19.0 (IBM Corporation, Armonk, NY, USA).

## 3. Results

### 3.1. 3PCA Ameliorates TNBS-Induced Colitis 

**Figure 1:**
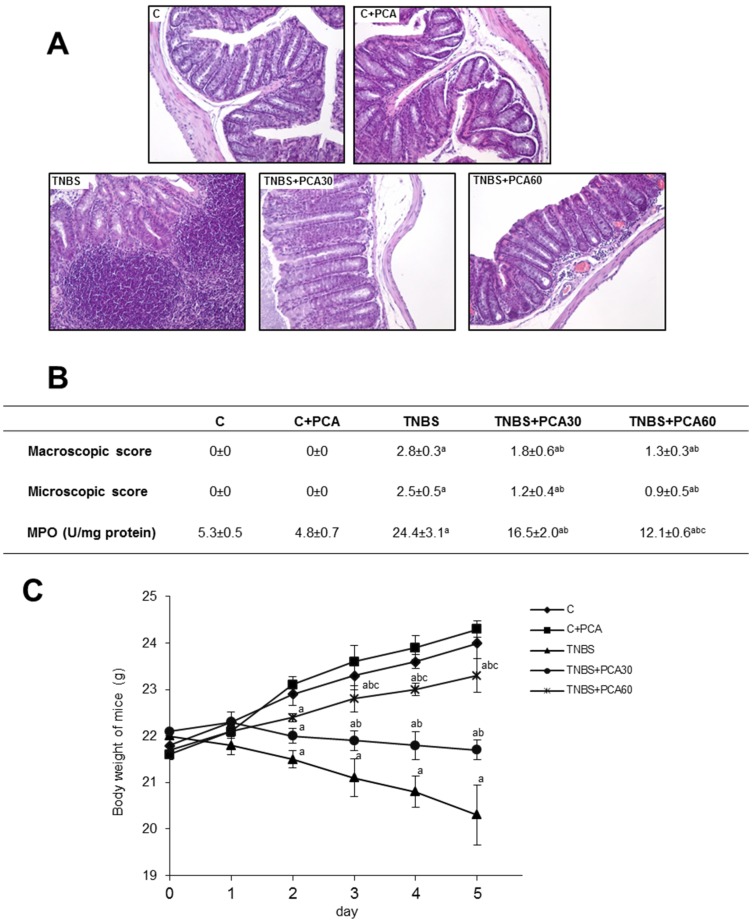
Effect of treatment with protocatechuic acid (PCA) on histological damage, macroscopic and microscopic scores, myeloperoxidase (MPO) activity and body weight in mice with 2,4,6-trinitrobenzenesulfonic acid (TNBS)-induced colitis. Representative sections of colonic tissue: control (C), C + PCA, TNBS, TNBS + PCA30 and TNBS + PCA60, stained with hematoxylin-eosin. Photographs are taken under magnification ×200. Ten non-overlapping randomly chosen fields of each section were scored (**A**). Macroscopic and microscopic scores and myeloperoxidase (MPO) activity (**B**), and body weight (**C**). Data are expressed as the means ± SEM of six mice per group. ^a^ *p* < 0.05 vs. C group; ^b^ *p* < 0.05 vs. TNBS group; ^c^ *p* < 0.05 vs. TNBS + PCA30 group.

The therapeutic efficacy of PCA on experimental colitis was assessed by macroscopic and microscopic damage of the colonic mucosa, body weight change and MPO activity. Colitis was induced by intracolonic administration of 2,4,6-TNBS. TNBS-treated animals exhibited a severe inflammation of the colon five days after rectal administration characterized by thickening of the mucosa, extensive destruction of mucosal epithelium with necrotic areas, submucosal edema and ulcers on the mucosal surface (Figure 1). The macroscopic and histological features of the colons from untreated control mice and control mice receiving PCA were normal. Severe macroscopic damage of the colon was observed at day five after TNBS rectal administration, however the macroscopic damage was significantly lower in mice received PCA at both doses reducing the presence of mucosal edema and hemorrhagic ulcerations. The histopathological features (range 0–3, indicated in Materials and Methods) of TNBS-treated mice included crypt destruction and submucosal edema characterized by inflammatory cell infiltration in mucosa and submucosa. Animals treated with PCA had decreased inflammatory infiltrate. PCA was also effective to alleviate ulcer severity and total colitis score in comparison to the TNBS group (Figure 1A,B). 

The beneficial effects of PCA observed macroscopically and microscopically were also evidenced biochemically. TNBS promoted an intense neutrophil infiltration, as revealed by the higher colonic MPO activity observed in TNBS-treated mice in comparison with the healthy control group (Figure 1B). The enzyme activity was significantly reduced in a dose-dependent manner after treatment with both PCA doses, revealing the decrease of neutrophil infiltration that takes place in the inflamed tissue. TNBS-induced colitis caused a progressive decrease of body weight in mice, which was significantly prevented by both doses of PCA in a dose-dependent manner (Figure 1C).

### 3.2. PCA Attenuates Oxidative Stress and Increases Antioxidant Enzyme Expression

**Figure 2:**
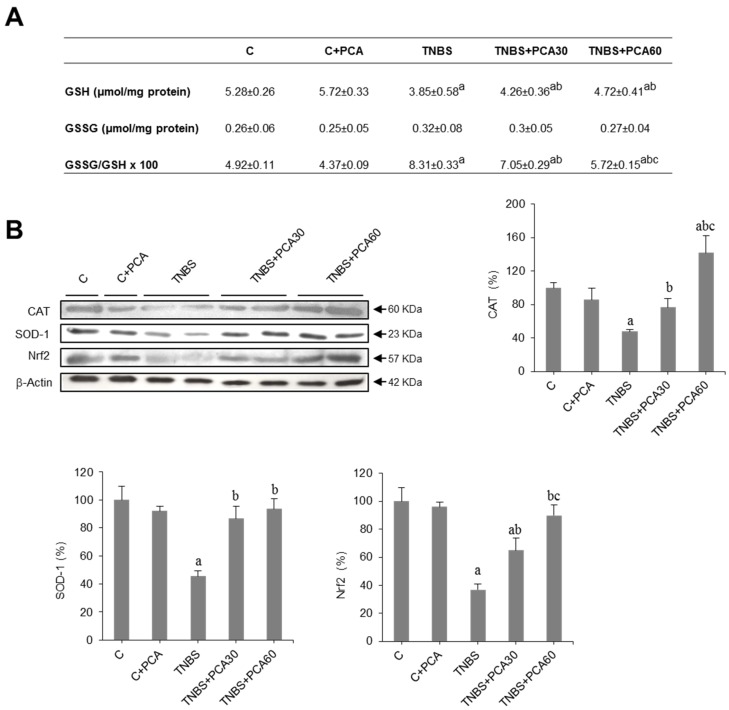
Effect of treatment with protocatechuic acid (PCA) on antioxidant status in mice with 2,4,6-trinitrobenzenesulfonic acid (TNBS)-induced colitis. Glutathione levels and GSSG/GSH ratio (**A**). Representative blots for CAT, SOD-1 and Nrf2 proteins, and results of densitometric quantification. (**B**). Values are expressed as the means ± SEM of six mice per group. ^a^ *p* < 0.05 vs. C group; ^b^ *p* < 0.05 vs. TNBS group; ^c^ *p* < 0.05 vs. TNBS + PCA30 group. Each assay was performed in triplicate.

We measured glutathione levels (GSH and GSSG) in colonic tissue. GSH concentration decreased significantly in the colon of mice receiving TNBS as compared to controls and this effect was significantly prevented in PCA-treated animals (Figure 2A). As a consequence, the GSSG/GSH ratio, a marker of oxidative stress, increased in the TNBS group and was significantly lower in colonic mice receiving PCA (Figure 2A). The decrease in the protein concentration of the antioxidant enzymes superoxide dismutase (SOD) and catalase (CAT) induced by TNBS was also significantly prevented by PCA administration (Figure 2B). Finally, we examined the involvement of nuclear factor-erythroid 2-related factor 2 (Nrf2) in the regulation of antioxidant enzyme genes. Data obtained indicate that Nrf2 protein levels decreased in colon of the TNBS group and this effect was prevented in mice receiving PCA (Figure 2B).

### 3.3. PCA Downregulates Expression of Proinflammatory Mediators

**Figure 3:**
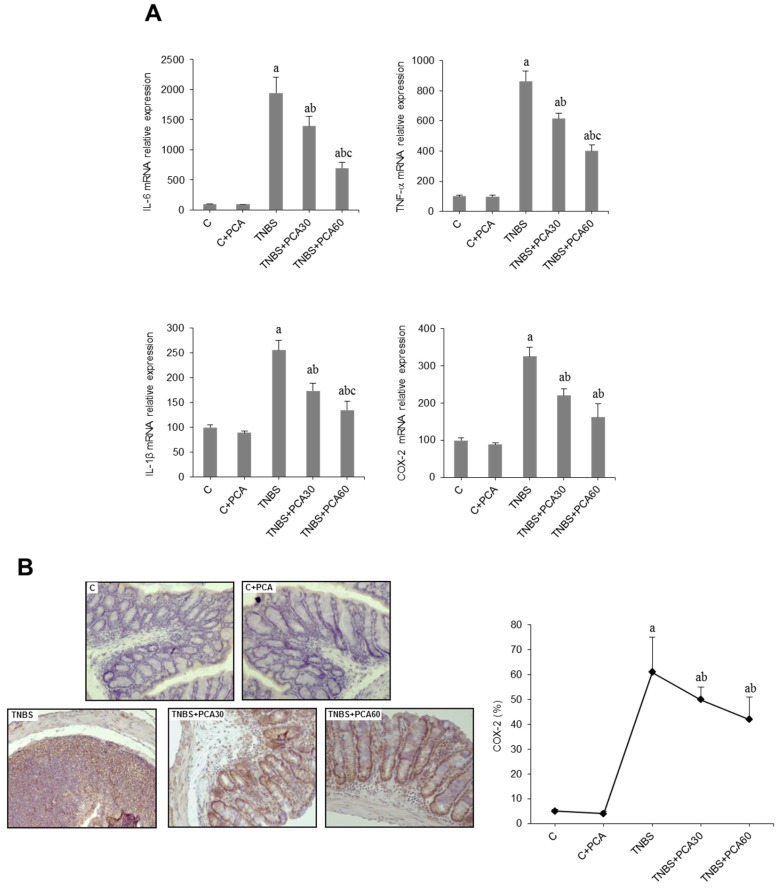
Effect of treatment with protocatechuic acid (PCA) on inflammation-related genes in mice with 2,4,6-trinitrobenzenesulfonic acid (TNBS)-induced colitis. mRNA expression of IL-6, TNF-α, IL-1β and COX-2 genes (**A**). Representative sections of COX-2 immunohistochemistry in colonic tissues from C, C + PCA, TNBS, TNBS + PCA30 and TNBS + PCA60, and quantification of the positive cells. Image analysis was performed using the ImageJ software v3.91 with ten non-overlapping randomly chosen histological fields. Photographs are taken under magnification ×200 (**B**). Data are expressed as the means ± SEM of six mice per group. ^a^ *p* < 0.05 vs. C group; ^b^ *p* < 0.05 vs. TNBS group; ^c^ *p* < 0.05 vs. TNBS + PCA30 group.

The anti-inflammatory mechanisms are of crucial importance to the homeostasis and integrity of the bowel. We investigated whether the antioxidant effect of PCA was linked to changes in the expression of inflammatory mediators. Compared with the control group, the expression of IL-6, TNF-α, IL-1β and cyclooxygenase-2 (COX-2) increased markedly in the colon of TNBS mice (Figure 3A). Data obtained revealed a significant decrease in the RNA levels of those inflammatory markers following PCA administration (Figure 3A). The anti-inflammatory effect of PCA was further confirmed by COX-2 immunostaining. As shown in Figure 3B, only low levels of COX-2 were detected in tissue of control mice, but these values were markedly increased following TNBS administration. PCA administration significantly reduced immunohistochemical expression of COX-2 in the colon tissue of TNBS-treated mice. 

### 3.4. PCA Modulates SphK/S1P and Related Signaling Pathways 

**Figure 4:**
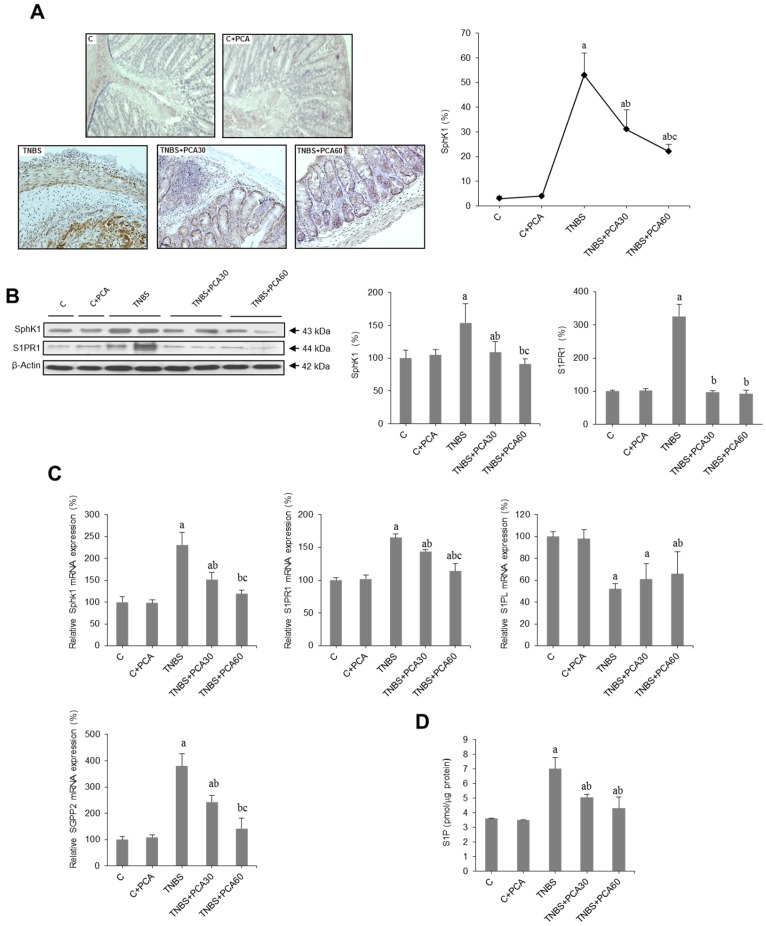
Effect of treatment with protocatechuic acid (PCA) on SphK/S1P signaling pathway-related genes in mice with 2,4,6-trinitrobenzenesulfonic acid (TNBS)-induced colitis. Representative sections of SphK1 immunohistochemistry in colonic tissues from C, C + PCA, TNBS, TNBS + PCA30 and TNBS + PCA60, and quantification of the positive cells. Image analysis was performed using the ImageJ software v3.91 with ten non-overlapping randomly chosen histological fields. Photographs are taken under magnification ×200 (**A**). Representative blots for SphK1 and S1PR1 protein, and results of densitometric quantification (**B**). mRNA expressions of SphK1, S1PR1, S1PL and SGPP2 genes (**C**), and level of S1P in colon homogenates analyzed by ELISA (**D**). Values are expressed as the means ± SEM of six mice per group. ^a^ *p* < 0.05 vs. C group; ^b^ *p* < 0.05 vs. TNBS group; ^c^ *p* < 0.05 vs TNBS + PCA 30 group. Each assay was performed in triplicate.

To investigate whether PCA ameliorates colonic damage in mice with TNBS-induced colitis through the regulation of the SphK/S1P axis, we first examined the expression of SphK1 by qRT-PCR, Western blot and immunohistochemistry. Both mRNA levels and protein concentration of SphK1 increased significantly in the TNBS group. These effects were prevented by PCA administration in a dose-dependent manner (Figure 4B,C). Data were confirmed by SphK1 immunostaining, with an increase in the percentage of SphK1 positive cells in colitic animals that was prevented by PCA (Figure 4A). Furthermore, we measured S1P levels by an ELISA test, which revealed a significant increase in mice treated with TNBS that was significantly abrogated by PCA (Figure 4D). Regarding the S1PR1 receptor both mRNA level and protein concentration were upregulated in colitic animals and this effect was prevented by PCA (Figure 4B,C). Importantly, expression of S1P lyase (S1PL), the only enzyme that can irreversibly degrade S1P, was markedly diminished by TNBS treatment and increased following PCA administration (Figure 4C). On the contrary, PCA prevented in a dose-dependent manner the increase in the expression of S1P phosphatase (SGPP)2, which promotes disruption of mucosal integrity (Figure 4C). 

**Figure 5:**
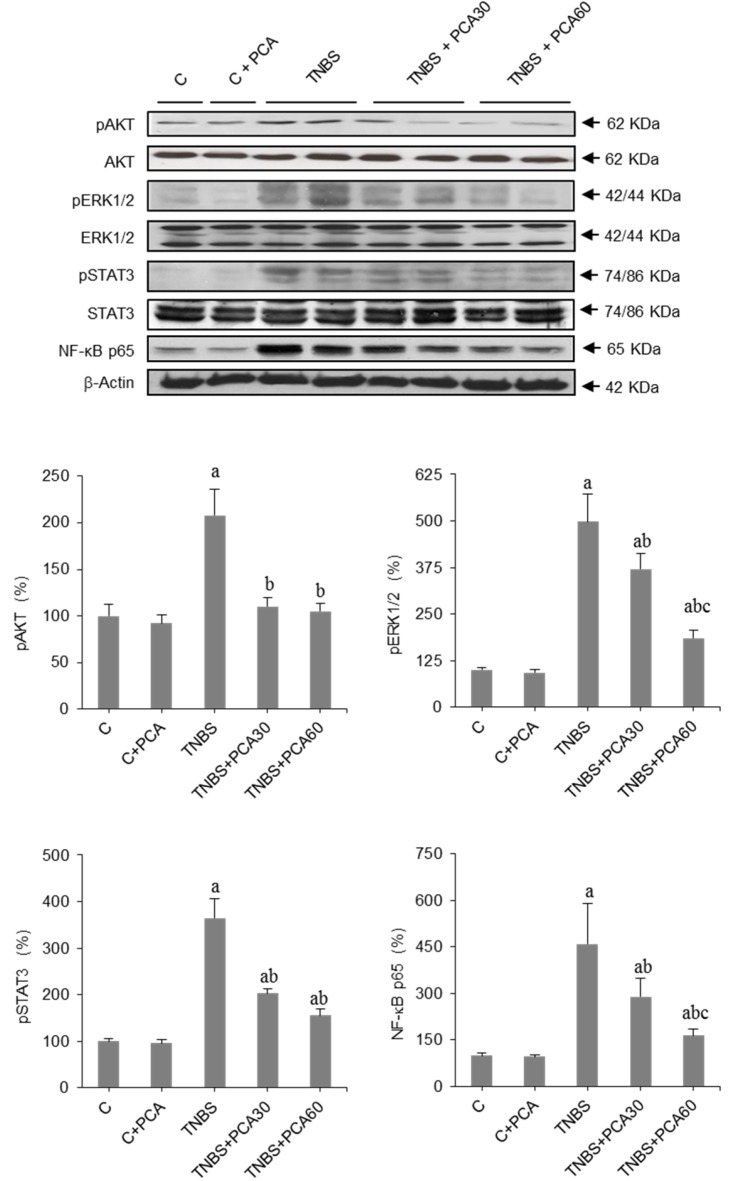
Effect of treatment with protocatechuic acid (PCA) on AKT, ERK, STAT and NF-κB expression in mice with 2,4,6-trinitrobenzenesulfonic acid (TNBS)-induced colitis. Representative blots for pAKT, AKT, pERK1/2, ERK, pSTAT3, STAT3 and NF-κB p65 proteins, and results of densitometric quantification. Values are expressed as the means ± SEM of six mice per group. ^a^ *p* < 0.05 vs. C group; ^b^ *p* < 0.05 vs. TNBS group; ^c^ *p* < 0.05 vs. TNBS + PCA30 group. Each assay was performed in triplicate.

Moreover, ShpK/S1P signaling has been implicated in the activation of STAT3 and NF-κB, which regulate the transcription of gene targets involved in the pathophysiology of IBD. To address whether the STAT3 pathway is involved in PCA driven events, we next evaluated the amount of phosphorylated and total STAT3 by Western blot analysis. Treatment with TNBS induced a marked increase in the phosphorylation of STAT3 that was significantly abrogated by PCA (Figure 5). NF-κB activation was measured by changes in the protein concentration of its p65 subunit, revealing an increased expression in colitic mice that was prevented by PCA treatment to a greater extent in the high dose group (Figure 5). Finally, we investigated changes in the PI3K/AKT and ERK pathways, which are involved in the inflammatory response mediated by NF-κB and STAT3 signaling. Data obtained demonstrate that TNBS induced a significant increase in the phosphorylation of AKT and ERK. These effects were significantly abrogated by PCA (Figure 5).

## 4. Discussion

IBD is a chronic disorder of the gastrointestinal tract characterized by exacerbated uncontrolled intestinal inflammation that represents an important worldwide public health problem. Although molecular mechanisms involved in the pathogenesis of IBD are still poorly understood, different studies have shown that the SphK/S1P pathway is activated in human IBD colons and in rodent models of IBD and its inhibition may prove to be a valuable therapeutic target [5,6,19]. The aim of this in vivo study was to explore if beneficial effects of PCA in an experimental model of IBD were associated to modulation of the SphK/S1P and related signaling pathways.

In a recent research in which the PCA was administered to rats with DSS-induced ulcerative colitis [12], the phenolic acid was given in drinking water from five days before initiating the oral administration of DSS. In spite of the difficulty to determine the precise dosage of PCA, and with the limitation that only a preventive effect before the onset of colonic damage could be demonstrated, it was found that the phenolic compound improved significantly the oxidative status and reduced the plasma increase of pro-inflammatory cytokines in colitic animals. In the present study, in which PCA was administered intraperitoneally, beginning simultaneously with the induction of IBD by intracolonic administration of TNBS in mice, colonic damage was attenuated and results obtained confirm the antioxidant and immunomodulatory properties of PCA. Thus, the phenolic acid inhibited oxidative stress induced by TNBS, increasing the protein concentration of antioxidant enzymes. In parallel, PCA enhanced the expression of the transcription factor Nrf2, an important regulator of the cellular antioxidant response through antioxidant response element-mediated induction of diverse antioxidant enzymes [20]. Moreover, expression of different pro-inflammatory cytokines and COX-2 was significantly reduced by administration of the phenolic acid, and there was a decrease of colonic MPO activity, confirming the ability of PCA to abrogate neutrophil infiltration. Antioxidant and anti-inflammatory effects of PCA have also been shown in other animal models of disease, such as streptozotocin-induced diabetes, ischemia reperfusion or hepatic steatosis [21].

Previous reports have demonstrated the preventive and therapeutic effect of polyphenols in animal models of IBD [22,23,24]. Different mechanisms have been proposed for their intestinal anti-inflammatory activity, such as antioxidant and immunomodulatory properties, effects on intestinal barrier function or interaction with gut microbiota [7]. However, only one study has attributed the anti-inflammatory properties of resveratrol in rats with ulcerative colitis induced by oxazolone to inhibition of SphK1, whose activity correlated with the disease activity index [8]. Data from the present study indicate that S1P levels increased in TNBS-treated mice, confirming previous findings in mice with DSS-induced colitis [19], while PCA treatment resulted in a lowered production of S1P.

Intracellular S1P levels are tightly maintained by the balance between synthesis and degradation. S1P is formed by phosphorylation of sphingosine in a process catalyzed by two distinct SphKs, and once formed may be irreversible degraded by S1PL to hexadecenal and phosphoethanolamine [25]. SphK1 is overexpressed in colon of IBD patients and in mice with DSS-induced colitis, and mice lacking SphK1 are less susceptible to experimentally induced IBD [26]. On the other hand, S1PL is highly expressed in enterocytes but downregulated in intestinal biopsy samples from patients with ulcerative colitis or in colon cancer [5,6]. Our data confirm the inverse relationship between the expression of SphK1 (increased) and S1PL (decreased) in TNBS-treated animals, and how both effects were abrogated by PCA administration, thus contributing to the reduction of S1P levels. Results are consistent with recent studies showing that SphK inhibitors significantly abrogate colitic damage and reduce the expression of IL-6 and COX-2 [27], and with the enhancement of colitis severity and inflammation by deletion of S1PL in DSS mouse models [5]. 

Effects of PCA on the SphK/S1P axis were associated to a lower expression of the S1PR1 receptor, which is considered a novel therapeutic target in ulcerative colitis [26]. It is known that upregulation of SphK1 expression, formation of S1P, and subsequent activation of S1PR1 play an essential role in maintaining persistent activation of the critical transcription factors NF-κB and STAT3 in a malicious feed-forward amplification loop linked to TNF-α and IL-6 that leads to chronic intestinal inflammation and carcinogenesis [28,29]. Clinical studies have revealed that expression of TNF-α and IL-6 and activation of NF-κB and STAT3 are increased in patients with active UC and particularly in those who progress to colorectal cancer [30,31]. Upregulation of those factors has also been reported in DSS or TNBS-animal models of colitis [32,33,34,35]. Treatment with the S1P receptor antagonist FTY720/fingolimod suppresses the amplification loop that leads to NF-κB and STAT3 activation [29]. Moreover, inhibitors of SphK such as ABC747080 or ABC294640 prevent TNF-α activation of NF-κB and reduce DSS-induced damage in mice [36]. In our study, PCA treatment significantly reduced the activation of both transcription factors in parallel with the modulatory effects on the SphK/S1P axis, thus supporting the usefulness of strategies involving the inhibition of sphingolipid signaling. Effects may, in turn, be related to the inhibition of the PI3/AKT and ERK pathways induced by the phenolic acid, because S1PR1 couples exclusively to heterotrimeric Gi to activate these kinases [37]. TNF-α-induced phosphorylation of AKT is significantly suppresses in HT-29 colonic epithelial cells by 7-*O*-succinyl macrolactin A in parallel to inhibition of NF-κB translocation [38]. In murine models of DSS *Rosmarinus officinalis* extract ameliorates intestinal inflammation through MAP kinases/NF-κB signaling [39]. It is also known that expression of the novel adipokine CTRP4 suppresses colitis in mice with a marked downregulation of ERK, AKT and STAT3 [40]. Abrogation of the upregulation of the upstream kinases AKT and ERK could therefore play a role in the SphK/S1P-mediated anti-inflammatory effects of PCA.

Despite the fact that S1PL alone is responsible for regulating steady-state cellular, tissue and extracellular S1P levels that S1P is a substrate for many lipid phosphatases [2]. Although little is known about the physiological function of SGPPs, it has been reported that SGPP2 promotes disruption of mucosal integrity and contributes to ulcerative colitis in mice and humans, being a therapeutic target in IBD [41]. An interesting finding from our study was that PCA abrogated the marked increase of SGPP2 expression observed in colitic mice. This effect could in turn be related to changes induced by the phenolic compound on the NF-κB pathway, because SGPP2 expression is a gene regulated by the transcription factor whose promoter has NF-κB motifs [42].

## 5. Conclusions

In conclusion, our data support the protective role of PCA on TNBS-induced colonic damage and show that the anti-inflammatory effect of the phenolic compound associates to an abrogation of the SphK/S1P axis and the related NF-κB and STAT3 signaling pathways with which it interacts. Although the exact role of the SphK/S1P system in regulating the pathogenesis of IBD still remains to be fully elucidated, present findings reinforce the interest in novel molecular pathways that may represent an attractive target for therapeutic interventions in IBD.

## Abbreviations

CAT	catalase
COX-2	cyclooxygenase-2
GSH	reduced glutathione
GSSG	oxidized glutathione
IBD	inflammatory bowel disease
IL	interleukin
MPO	myeloperoxidase
NF-κB	nuclear factor kappa B
Nrf2	nuclear factor-erythroid 2-related factor 2
PCA	protocatechuic acid
S1P	sphingosine-1-phosphate
S1PL	S1P lyase
SGPP	S1P phosphatase
S1PR	S1P receptor
SOD	superoxide dismutase
SphK	sphingosine kinase
STAT	signal transducer and activator of transcription
TNBS	2,4,6 trinitrobenzenesulfonic acid
TNF	tumor necrosis factor
