# Correction: Crespo et al. Protective Effect of Protocatechuic Acid on TNBS-Induced Colitis in Mice Is Associated with Modulation of the SphK/S1P Signaling Pathway. *Nutrients* 2017, *9*, 288

**DOI:** 10.3390/nu16060774

**Published:** 2024-03-08

**Authors:** Irene Crespo, Beatriz San-Miguel, José Luis Mauriz, Juan José Ortiz de Urbina, Mar Almar, María Jesús Tuñón, Javier González-Gallego

**Affiliations:** 1 Institute of Biomedicine (IBIOMED), University of León, 24071 León, Spain; icreg@unileon.es (I.C.); bsanv@unileon.es (B.S.-M.); jl.mauriz@unileon.es (J.L.M.); malmg@unileon.es (M.A.); mjtung@unileon.es (M.J.T.); 2 Centro de Investigación Biomédica en Red de Enfermedades Hepáticas y Digestivas (CIBERehd), 24071 León, Spain; 3 Pharmacy Service, Complejo Asistencial Universitario de León, 24071 León, Spain; jortiz@saludcastillayleon.es

In the original publication [1], there was a mistake in Figure 5, as published. The STAT3 representative blot was wrong due to an unintentional error in the selection of the image during the editing process. The corrected Figure 5 appears below. The authors apologize for any inconvenience caused and state that the scientific conclusions are unaffected. This correction was approved by the Academic Editor. The original publication has also been updated.

## Figures and Tables

**Figure 5:**
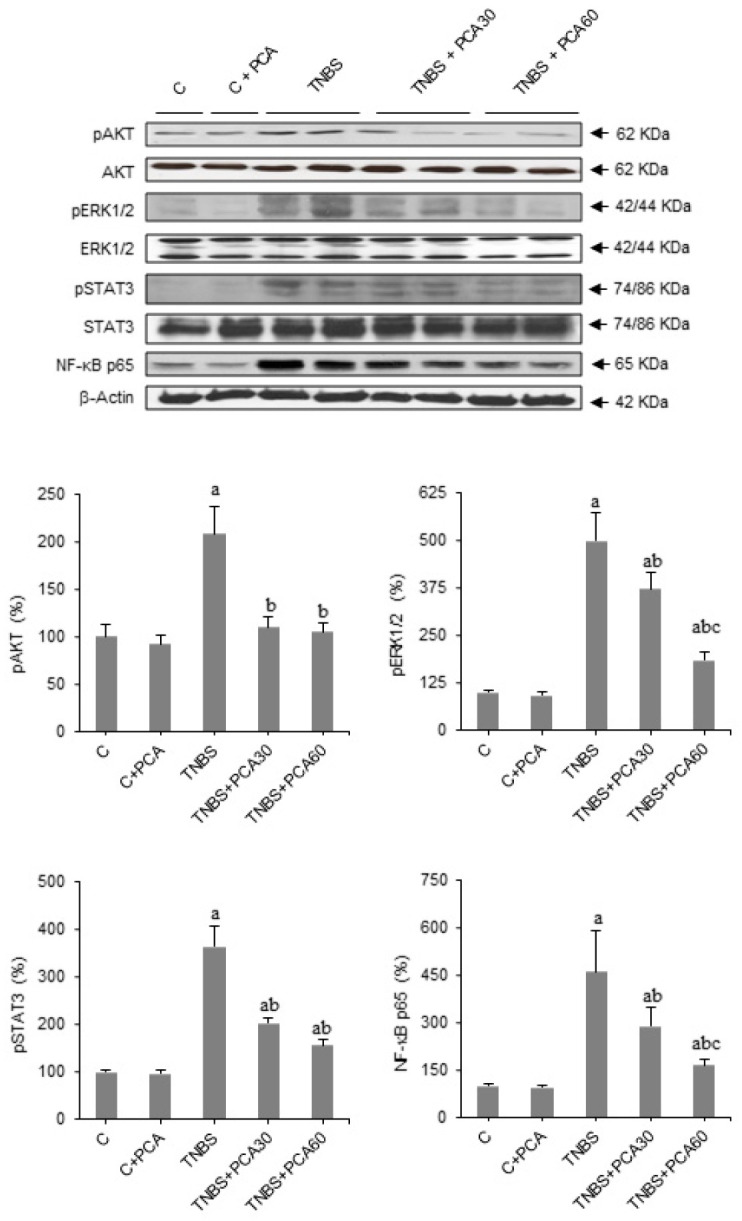
Effect of treatment with protocatechuic acid (PCA) on AKT, ERK, STAT and NF-κB expression in mice with 2,4,6-trinitrobenzenesulfonic acid (TNBS)-induced colitis. Representative blots for pAKT, AKT, pERK1/2, ERK, pSTAT3, STAT3 and NF-κB p65 proteins, and results of densitometric quantification. Values are expressed as the means ± SEM of six mice per group. ^a^ *p* < 0.05 vs. C group; ^b^ *p* < 0.05 vs. TNBS group; ^c^ *p* < 0.05 vs. TNBS + PCA30 group. Each assay was performed in triplicate.
